# 

**Published:** 1907-03-09

**Authors:** 


### BOOKS RECEIVED.

Cornish Bros.

"Insanity Cured by a New Treatment." By C. W. Suckling, M.D.

Methuen and Co.

"The Drink Problem." Edited by T. N. Kelynack, M.D.

### THE NURSES' BOOKSHELF.

### MEDICAL ELECTRICITY AND THE LIGHT TREATMENT.

By Kate Neale, Sister in Charge Light Department, Guy's Hospital. Illustrated.
2/6 net, 2/9 post free.

### ELEMENTARY TREATISE ON THE LIGHT TREATMENT FOR NURSES.

By James H. Sequeira, M.D. Lond. Illustrated.
2/6 net, 2/9 post free.

### ART OF FEEDING THE INVALID.

By a Medical Practitioner and a Lady Professor of Cookery.

New and Popular Edition, with Special Chapter on Feeding in Consumption. 1/6 post free.

### DIET IN SICKNESS AND IN HEALTH.

By Mrs. Ernest Hart. With an Introduction by Sir Henry Thompson, F.R.C.S., M.B. Lond.
3/6 post free.

### LECTURES UPON THE NURSING OF INFECTIOUS DISEASES.

By F. J. Woollacott, M.A., M.D.
2/6 net, 2/9 post free.

### HINTS TO NURSES ON TROPICAL FEVERS.

By S. F. Pollard, Sister Army Nursing Reserve, late Sister Guy's Hospital.
The Work is based upon the Personal Experience of the Author Abroad
1/6 net, 1/8 post free.

### NURSING IN DISEASES OF THE THROAT, NOSE, AND EAR.

By P. Macleod Yearsley, F.R.C.S. Eng., M.R.C.S., L.R.C.P. Lond. Illustrated.
2/6 post free.

### OPHTHALMIC NURSING.

By Sydney Stephenson, M.D., F.R.C.S.E.
New and Revised Edition, profusely Illustrated, and a Glossary of Terms.
3/6 net, 3/10 post free.

### THE MODERN NURSING OF CONSUMPTION.

By Dr. Jane H. Walker.
1/- net, 1/2 post free.

### OUTLINES OF INSANITY.

**A Popular Treatise on the Salient Features of Insanity.**

By Francis H. Walmeley, M.D. Popular Edition, stitched. 1/6 post free.

### SIMPLE INTRODUCTORY LESSONS ON MIDWIFERY.

By M. Loane. Illustrated.
1/- net, 1/1 post free.

### INSTRUCTIONS TO MIDWIVES.

Compiled by the Midwives Supervising Committee of the Corporation of Manchester.
6d. net, 7d. post free.

### SIMPLE SANITATION:

**The Practical Application of the Laws of Health to Small Dwellings.**

By M. Loane. With Introductory Chapter on Sanitary Legislation by D. A. Mearns Proser. 1/- net, 1/2 post free.

### LECTURES ON HOME NURSING FOR THE POOR.

By a District Nurse. Illustrated.
1/6 net, 1/8 post free.

### THE CARE OF CHILDREN.

**Practical Hints for Mothers and Nurses at Home and Abroad.**

By Robt. J. Blackham, D.P.H. Lond.
Revised and Enlarged Edition.
1/6 net, 1/8 post free.

### THE HUMAN BODY:

**Its Personal Hygiene and Practical Physiology.**

By B. P. Colton, Professor of Natural Science, Illinois University. Profusely Illustrated.
5/- post free.

*Catalogue of Nursing Manuals sent post free on application.*

### The Scientific Press, Ltd.,

**Publishers of Nursing Text-Books, Charts, and Case-Books of all Descriptions.**

**28 & 29 SOUTHAMPTON STREET, STRAND, LONDON, W.C.**

